# Urethral length and its relationship with anthropometric parameters in adult male Nigerians

**DOI:** 10.1186/s12894-023-01360-0

**Published:** 2023-11-15

**Authors:** Rufus Wale Ojewola, Moses Adebisi Ogunjimi, Emmanuel Abayomi Animashaun, Michael Olatunji Afolayan, Sixtus Ogbonnia Osi, Emmanuel Ajibola Jeje, Kehinde Habeeb Tijani

**Affiliations:** 1 Department of Surgery, Faculty of Clinical Sciences, College of Medicine of University of Lagos, Idi-Araba, Surulere, Lagos, Nigeria; 2 Urology Unit, Department of Surgery, Lagos University Teaching Hospital, Idi-Araba, Surulere, Lagos, Nigeria

**Keywords:** Urethral length, Anthropometric parameters, Age, Race

## Abstract

**Background:**

The available literature on urethral length in adult males is limited. To the best of our knowledge, such measurement has never been carried out amongst Nigerian and African men. This study aimed to document average urethral length as well as the relationship between urethral length and age, and anthropometric parameters amongst the Nigerian male adult population to add to the database for urethral length.

**Methods:**

It was a prospective cross-sectional study amongst adult male Nigerians who had an indication for urethral catheterization as part of their regular treatment at the urologic clinics as well as male adult patients who required urethral catheterization in the theatre and the wards for various reasons. All patients had anthropometric parameters measured or calculated. The total usable length (A) of the catheter was measured before catheterization and the unused catheter length (B) was measured after catheterization for each patient. The calculated urethral length (C) was obtained by subtracting B from A. Data were analyzed using SPSS version 26.0. Mean urethral length was determined while the correlation between urethral length and age as well as anthropometric parameters were determined using Pearson Correlation.

**Results:**

A total of 450 adult males were recruited. The mean age of subjects was 63.58 years with a range of 22–91 years. The average total usable and unused catheter lengths were 30.01 and 8.97 cm respectively. The mean urethral length among participants was 21.32 cm (8.4 Inches) with a range of 16.5 to 28 cm (6.5–11.0 Inches). There were no statistically significant correlations between urethral length and age ([r (450) = − 0.029, *p* = 0.546]) as well as with anthropometric parameters (height: r (450) = − 0.088, *p* = 0.61; weight: [r (450) = − 0.047, *p* = 0.324 and BMI: r (450) = − 0.082, *p* = 0.08) in adult males.

**Conclusion:**

This study suggests that there may be racial differences in adult male urethral length but no relationship with age and anthropometric parameters. Further research is needed to explore these findings.

## Background

The male urethra plays a critical role in the transport of urine and semen during ejaculation. Being the only part of the urinary tract that communicates with the exterior, it also constitutes a crucial access for the instrumentation of the male urinary tract, both lower and upper, for both diagnostic and therapeutic purposes.

The length of the urethra can vary from person to person, and several factors can affect its length. The range of urethral length in adult men has been quoted from various sources to be about 18-25 cm [1]. Sir Henry Gray had measured the cadaveric male urethra and documented a length of 18 to 20 cm, which continues to be a reference standard quoted by most authors to date [2]. Since then, there has been a paucity of literature on this subject even at the international level [3, 4].

Age is another factor that can influence urethral length in males. In infants, the urethra is short, and as the body grows, the urethra lengthens. During puberty, the male body undergoes significant changes, including the growth of the penis, testicles, and urethra [5]. The urethra continues to grow until the age of 18–20 years, after which it remains relatively stable [6]. On the other hand, diseases of the urethra like urethral stricture diseases can reduce the urethral length considerably. The relationships between height, weight, body mass index (BMI) and urethral length are equivocal and there is limited research on the effect of race on urethral length in males. However, there are some suggestions that there may be racial differences in penile and therefore urethral length [7].

In our practice, we encountered difficulties in performing transurethral resection of the prostate in two patients within a short period because the resectoscope appeared shorter than the urethral length in these men. Hence, the inspiration to study urethral length in adult Nigerian men. To the best of our knowledge, the urethral length has not been measured in Nigerian or African adult male populations before. This study was an attempt to develop a simple anatomical database for urethral length and examine the relationship between urethral length and anthropometric parameters amongst the Nigerian male adult population.

## Methods

This prospective cross-sectional observational study was carried out at the Lagos University Teaching Hospital, Idi-araba, Lagos, Nigeria. The study population was adult male patients who required urethral catheterization as part of regular treatment at the urologic clinics as well as male adult patients who required urethral catheterization in the theatre and in the wards for various reasons. The study spanned two years from July 2020 and June 2022. Adult male patients on a catheter for urinary retention who were for routine catheter change were recruited in the clinic and patients in theatre undergoing both elective and emergency surgeries who required urethral catheterization for monitoring under anaesthesia. Others were male patients on the ward who required urethral catheterization for both urological and non-urological indications. Patients with a history of congenital or acquired urethral abnormalities, those who have had urethral and prostate surgeries, active urethral or prostatic infections and patients having an erection during anaesthesia or catheterization were excluded. Patients who did not consent to the study were also excluded.

The sample size (N) for the study was calculated using the formula to determine a sample size for estimating a population mean [8, 9];

$$N=\frac{\ Z\upalpha /2^{2}\kern0.5em x\kern0.5em \upsigma\ 2}{E^2}$$

Where;

Z = 1.96 at a confidence interval of 95%.

σ = standard of deviation which is 0.5 for the unknown population and.

 E = desired margin of error or precision set at 0.05.

 N = [1.96 × 0.5 /0.05] ^2^ = 384.16.

**Fig. 1:**
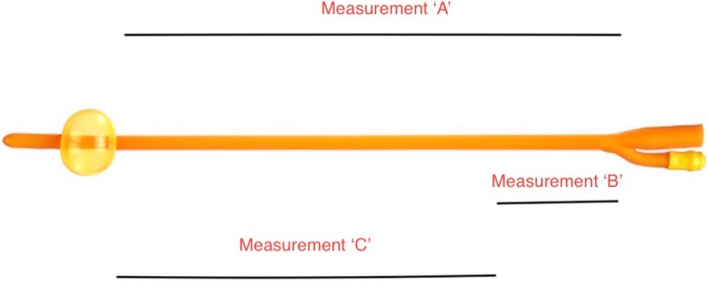
Foley catheter showing the measured and calculated lengths

For all recruited patients, anthropometric parameters including weight and height were measured and BMI was calculated. Following this, the balloon of a sterile Foley catheter was inflated with 10 cc of sterile water before insertion in a sterile field. The length from the balloon to the ‘Y’ junction was measured while the catheter was still in the inner sterile cover. This was documented in centimetres (measurement A). The balloon was then deflated and the catheter passed into the bladder using an aseptic technique, followed by the re-inflation of the balloon to the same volume of fluid (10 cc). The inserted catheter was pulled gently to ensure the balloon was at the bladder neck and the penis was gently straightened without stretching when necessary. The length of the catheter outside the penis was measured to the pre-marked point at the ‘Y’ junction (measurement B). Subtracting B from A gave the length of the urethra in each patient (measurement C). Figure 1 shows a Foley catheter with various measured or calculated lengths. All these processes were carried out in a sterile way.

Data were collected from these patients using the study pro forma and analysed using the IBM SPSS Statistics for Macintosh, version 26.0 (IBM Co., Armonk, New York, USA). Descriptive statistical analysis was performed. Pearson’s correlation was used to determine the relationship between urethral length and age as well as anthropometric parameters. Results were displayed in tables and charts.

## Results

**Fig. 2:**
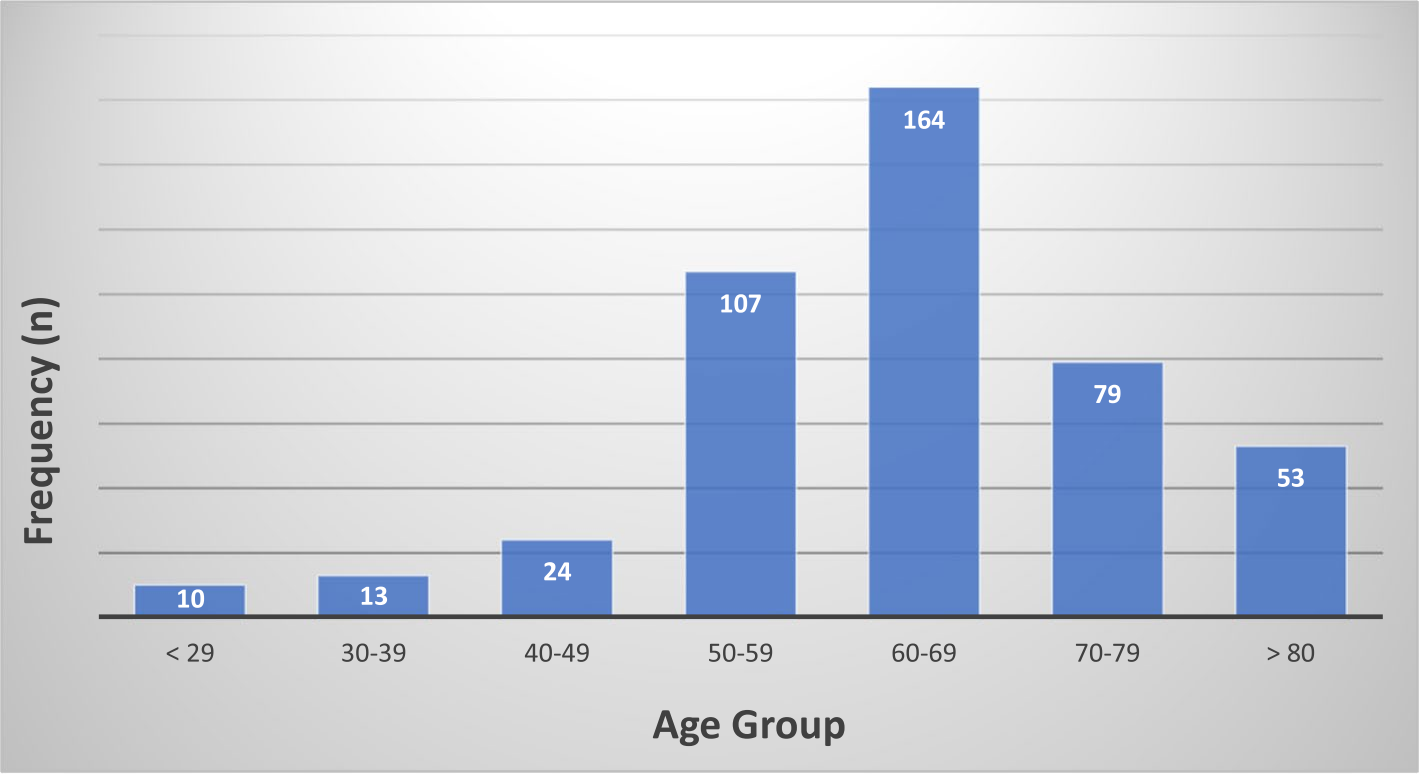
Age distribution of patients

A total of 450 adult male patients were recruited into the study between July 2020 and June 2022. The mean age of patients was 63.58 years with a standard deviation (SD) of 12.95 and a range of 22–91 years. The peak age range was 60–69 which constituted 36.4% of the study population. The age distribution of the participants is depicted in Fig. 2 below.

**Fig. 3:**
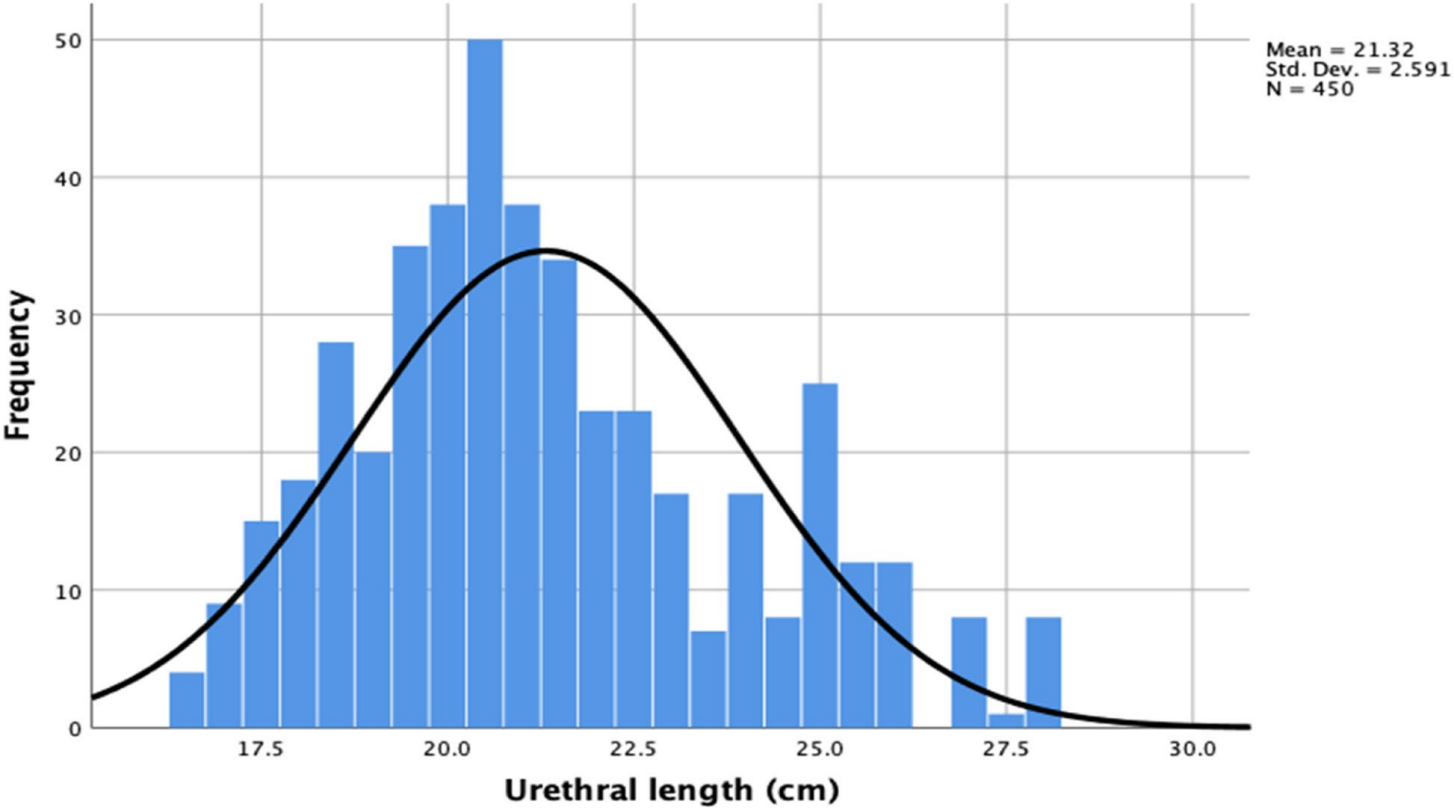
Histogram representing the normal distribution of urethral length

The average total usable length of the catheters was 30.01 cm while the average unused catheter length was 8.97 cm. The mean urethral length among participants was 21.32 cm (8.4 Inches) with a SD of 2.59 and a range of 16.5 to 28 cm (6.5 to 11.0 Inches). The statistical distribution of the data is reflected by the normal histogram on the frequency distribution curve in Fig. 3.

The mean weight, height and BMI of the participants were 67.14 kg; 1.66 m and 24.48 kg/m^2^ respectively. Other details are shown in Table 1 below.

**Table 1:** Descriptive statistics of patients (*n* = 450)

	Minimum	Maximum	Mean	SD
Age (years)	22.0	92.00	63.58	12.95
Weight (kg)	47.0	108.0	67.2	10.96
Height (m)	1.53	1.81	1.66	0.06
BMI (kg/m^2^)	16.23	44.54	24.51	4.02
Urethra(cm)	16.5	28.0	21.32	2.6

There was a non-significant negative correlation between urethral length and height [r (450) = −0.088, p = 0.61] and age [r (450) = − 0.029, p = 0.546] while there was a non-significant positive correlation between urethral length and weight [r (450) = − 0.047, *p* = 0.324] and BMI [r (450) = − 0.082, *p* = 0.08] in Nigerian male adult population. Table 2 below shows the average urethral length at different age ranges.

**Table 2:** Mean urethral length (cm) per age group

Age Group	n	Mean	SD	Min.	Max.	Range
20–29	10	20.070	0.6567	19.4	21.0	1.6
30–39	13	22.338	1.1095	20.3	23.5	3.2
40–49	24	21.275	3.1509	18.4	25.7	7.3
50–59	107	22.412	2.9097	16.5	28.0	11.5
60–69	164	20.683	2.4122	16.6	27.5	10.9
70–79	79	20.678	1.8329	17.0	23.3	6.3
≥ 80	53	22.045	2.6899	17.2	28.0	10.8

**Fig. 4:**
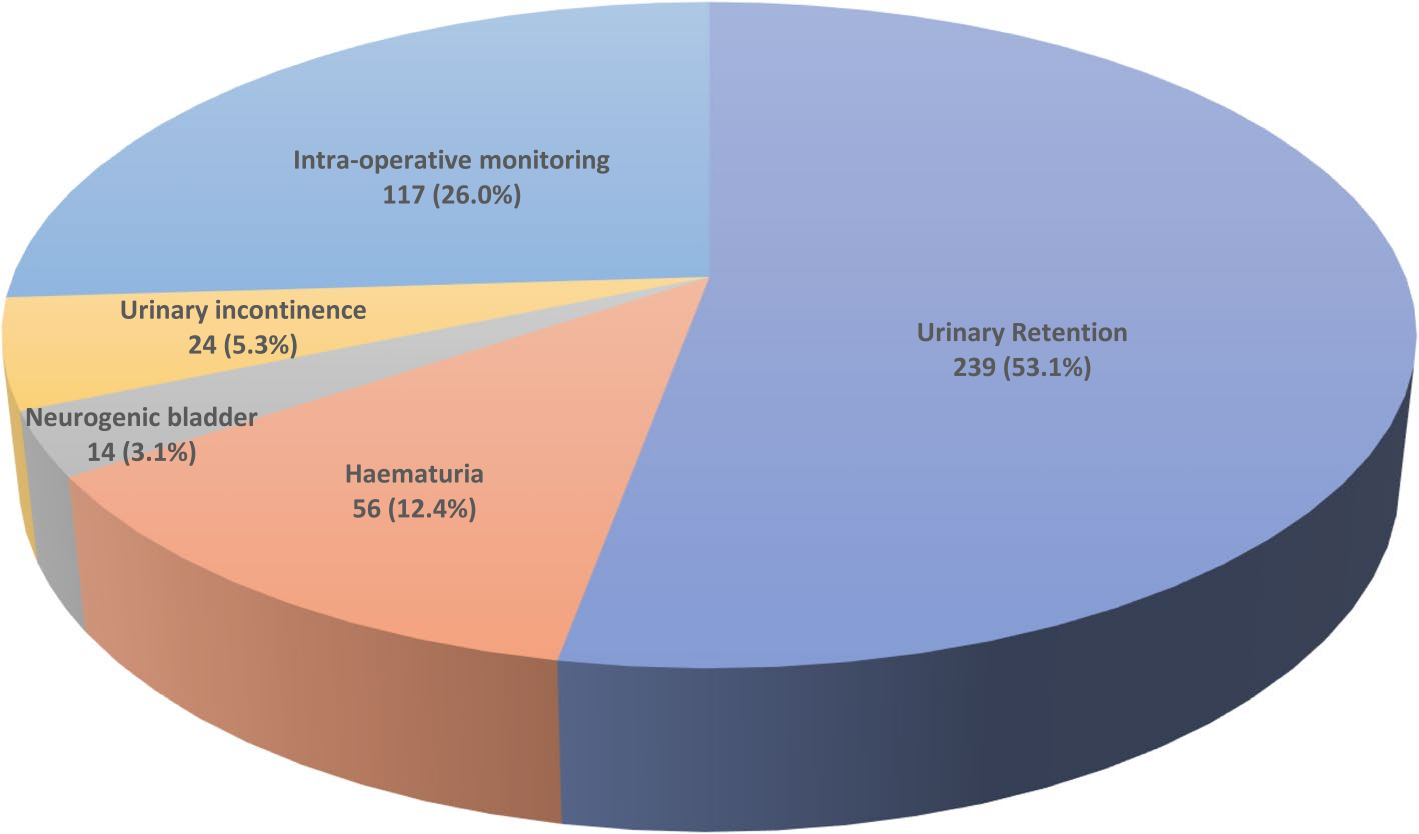
Indications for catheterization in 450 patients

Urinary retention was the most common indication for catheterization in 238 (51.8%) patients. Of these, 204 (85.4%) had retention due to benign prostatic hyperplasia and 35 (14.6%) secondary to prostate cancer. Other indications for urethral catheterization are depicted in Fig. 4 below.

## Discussion

Urethra is an important access to the urinary tract for urological procedures especially endourological procedures. Therefore, its health including length is of utmost importance in this era of rapid advancements in endourology. A good understanding of the length of the male urethra is crucial, as urologists sometimes rely on the calibration of the male urethra to evaluate the extent of a lesion or obstruction in the urethra [4]. Accurately assessing the normal length is therefore paramount, as it provides an idea of the location within the urethra. The length of the penis has little impact on the ability to access the prostate gland through the urethra, as the resectoscope can be manipulated to navigate around any anatomical structures. However, a longer urethra can make procedures like transurethral resection of the prostate or bladder tumour more challenging if the instrument is significantly shorter than the urethral length and may require specialized techniques or equipment like extra-long resectoscopes. The length of the urethra can therefore be important in planning treatment for medical conditions [4].

Despite its significance, there is limited data on the average length of the urethra in adult males generally and in different populations. With an extensive literature search, there is a scarcity of information on this subject as only two studies have documented urethral length in adult males before. One of the studies was amongst American men [3] while the other was amongst the Indian adult male population [4]. As none exists yet for Africans, this study aimed to provide additional information on this topic by measuring the urethral length in a sample of adult Nigerian males.

Various urethral and penile pathologies can affect the urethral length. Acquired pathologies such as urethral strictures and acquired hypospadias as well as congenital problems like hypospadias, epispadias and micropenis can reduce the available length of the urethra. Likewise, surgery like urethroplasty especially the anastomotic type where a segment of the urethra is excised will reduce the urethral length. Patients with these pathologies were excluded from our study so as not to distort our findings.

Several techniques are available for evaluating urethral length, but none have been established as the definitive standard. The initial approach to measuring urethral length involved direct dissection and measurement by Sir Henry Gray, resulting in a range of 18 to 20 cm. Although this method yields precise measurements, its limitation lies in the fact that it involves cadavers and this direct measurement is not practically feasible. Indirectly, urethral length can be measured using retrograde urethrogram and micturating cystourethrogram but the measurement is usually affected by the degree of magnification as well as the angle at which the X-ray focuses on the urethra. Magnetic Resonance Imaging (MRI) is usually used to evaluate lesions and diseases of the urethra but MR images do not capture the proximal sections of the prostatic urethra and distal penile urethra unless a Foley catheter is utilized for visualization [10]. This method is also expensive. A practical method is the use of a urethral catheter as described in this study. This method was utilized solely in the Indian study while most of the patients in the American study had this method used in their estimation. Alternatively, urethral length was established at the time of flexible cystoscopy upon removal of the cystoscope. The scope was held fixed at the bladder neck with the penis on stretch and the cystoscope was similarly marked with tape at the end of the penis. The cystoscope was then removed and the distance from the mark to the end of the cystoscope was measured in centimetres [3].

Our study of 450 subjects found that the average length of the urethra in adult Nigerian males is 21.3 cm ± 2.59 with a range of 16.5 to 28.0 cm. This is longer than the average urethral length of 17.55 cm reported amongst Indian adult men [4]. This translates to a difference of 3.69 cm in the urethral length between the two races. A possible explanation for this difference may be the age and pathology differences in the study population between these two studies. While the majority of the patients in our study had retention secondary to either benign prostatic enlargement or prostate cancer, the majority of patients in the Indian study were younger than 50 years of age and therefore with a lower incidence of prostatic diseases. Since prostatic enlargement will increase the length of the posterior urethral which also contributes to the total length of the urethra, this could have partially contributed to this difference. However, we do not think that this is sufficient to explain the difference as our data did not show any significant difference in urethral length between younger and older men in this present study. It might therefore mean that there is a racial difference in urethral length.

On the other hand, the urethral length in this study is slightly lower than reported by Kohler et al. [3] among American men where the average length was 22.3 cm which is approximately 1 cm longer than reported in our study. The exact reason for this difference may not be clear but a subtle difference in methodology might be contributory. In the American study, a gentle stretch was applied to the penis on the catheter before measurement on the catheter was undertaken [3]. In addition, subjectiveness like stretching may also distort the findings on measurement. Unlike what they did, we avoided stretching the penis in our patients before measurement because we believe that the stretching of the penis may lead to an overestimation of the urethral length because of the elastic nature of the penile tissue.

The total length of the urethra is made up of individual lengths of the prostatic, membranous, bulbar and penile urethra [1]. The differences in total urethral length may also result from differences in the penile length with corresponding differences in penile length between different races [11]. This study may be another one supporting Rushton’s theory that penis length is greatest in Negroids, smaller in Caucasoids and smallest in Mongoloids [11]. However, the figure documented in the work of Kohler et al. [3] did not support this theory as the length of the urethra was greater than reported in this study made up of the black population. Again, the stretched penile length used in that study might be a factor responsible for this amongst others. Furthermore, the study did not document the racial composition of the study group. These differences may, however, not have any significant clinical implications, but they could be valuable for understanding the anatomical differences between these populations. Further research may be needed to explore the potential factors contributing to these differences and their clinical implications.

From this study, the average usable length of most of the catheters currently in use in the Nigerian market is 30.01 cm. This is the length from the neck of the inflated balloon to the ‘Y’ junction of the catheter. This is at least one and a half times longer than the average length of the urethra obtained in this study. This means there is a need to perhaps customize the catheter and other instruments for our population. The average unused catheter length, i.e., the length between the tip of the penis and the ‘Y’ junction of the catheter, was 8.97 cm in this study. Customised catheters may be desirable to avoid wearing too long a catheter difficult or cumbersome to pack.

Our study did not reveal any significant relationship between the urethral length and age in adult male Nigerians. This further corroborates the findings in the study by Kohler et al. [3] which showed that there is no significant difference in urethral length between adult males of different ages. The growth and development of the male urethra occur primarily during foetal and childhood development [12]. It seems that the length of the urethra is primarily determined by genetic factors and is relatively consistent across individuals of the same race and ethnicity. It appears that once an individual reaches adulthood, the urethra is fully formed and is unlikely to change significantly in length.

Like other studies, there is no statistically significant correlation between urethral length and anthropometric parameters in our study. However, it should be noted that subjects in extremes of height, weight and BMI comprise only a small subset of the patients recruited in this study [3, 4]. The weight and BMI positively correlated while the height negatively with the urethral length but were not statistically significant. Again, the lack of a significant correlation between urethral length and BMI, height, or weight is likely because urethral length is primarily determined by genetic factors. While there may be slight variations in urethral length between individuals, this study has demonstrated that these variations are unlikely to be influenced by external factors such as BMI, height, or weight.

One of the limitations of this study is our inability to correct for varying degrees of intravesical prostatic protrusion as it affects the length of the urethral catheter taken up with urethral catheterization. Recruitment of only patients without prostatic enlargement or measurement of urethral length with a flexible cystoscope are options to overcome this limitation.

## Conclusion

This study suggests that there may be differences in male urethral length between different races. Our study also demonstrated that there is no correlation between urethral length and anthropometric parameters in adult male Nigerians. In addition, this study showed that there is no evidence to suggest that age has any significant impact on urethral length in adult males. While other factors such as genetics, race, and ethnicity can influence urethral length, age is not a significant contributing factor. Further research is needed to better understand the relationship between urethral length and anthropometric parameters in adult males and in different races.

## Data Availability

The datasets generated and analysed during the current study are available in the Mendeley data repository, [https://data.mendeley.com/datasets/kh39bmxc8j/1].
